# Preoperative risk stratification in endometrial carcinoma: a pragmatic, complementary nomogram using readily available clinical variables

**DOI:** 10.3389/fonc.2026.1894175

**Published:** 2026-09-22

**Authors:** Jiawen Fan, Yiwei Du, Bin Zhou

**Affiliations:** 1 Department of Radiology, The Affiliated Yangming Hospital of Ningbo University, Yuyao, Ningbo, Zhejiang, China; 2 Department of Gynecology, The Affiliated Yangming Hospital of Ningbo University, Yuyao, Ningbo, Zhejiang, China; 3 Department of Pathology, The Affiliated Yangming Hospital of Ningbo University, Yuyao, Ningbo, Zhejiang, China

**Keywords:** endometrial biopsy, endometrial carcinoma, MRI, nomogram, risk stratification

## Abstract

**Background:**

Preoperative differentiation between low-risk and non-low-risk endometrial carcinoma (EC) remains suboptimal using conventional assessment (endometrial biopsy combined with MRI); making this distinction is critical for tailoring surgery appropriately. This study aimed to develop a nomogram integrating conventional assessment with readily available variables, as a complementary tool to support risk reclassification and individualized surgical planning in selected clinical scenarios.

**Methods:**

A total of 251 patients with primary EC were retrospectively enrolled and divided into training (n = 179) and validation (n = 72) cohorts according to whether the last preoperative transvaginal ultrasound was performed at our institution or at external facilities. A nomogram was constructed using least absolute shrinkage and selection operator regression and multivariable logistic analysis. Model validation included 1,000 bootstrap resamples for optimism correction and Hosmer–Lemeshow testing for calibration, followed by internal validation in the independent validation cohort. Model performance was evaluated using the area under the curve (AUC), calibration slope, Brier score, and decision curve analysis (DCA). The ability of the nomogram to improve risk reclassification beyond conventional assessment was assessed with continuous net reclassification improvement (NRI) and integrated discrimination improvement (IDI).

**Results:**

The nomogram achieved an AUC of 0.902 (95% *CI*: 0.857–0.948) in the training cohort, with an optimism-corrected AUC of 0.892 after 1,000 bootstrap resamples. In the validation cohort, the AUC was 0.866 (*95% CI*: 0.773–0.959), calibration was satisfactory (slope, 0.810; Brier score, 0.142; Hosmer–Lemeshow *P* = 0.156), and the nomogram demonstrated comparable or superior net benefit to conventional assessment across the majority of threshold probabilities, and showed improved risk reclassification (continuous NRI, 1.189, *P* = 0.019; IDI, 0.126, *P* = 0.013).

**Conclusion:**

This nomogram, constructed from routinely available variables, demonstrated acceptable calibration in the validation cohort, despite a slight tendency toward risk underestimation. While the incremental AUC over conventional assessment was not statistically significant, the model showed favorable net benefit on DCA and improved risk reclassification, suggesting it may serve as a useful adjunct for risk reclassification in specific clinical settings. Whether this translates into measurable improvements in surgical decision-making and patient outcomes awaits confirmation in prospective multicenter studies.

## 1 Introduction

Endometrial carcinoma (EC) is among the most common gynecological malignancies worldwide. Over the past four decades, its incidence and mortality rates have shown a consistent upward trend, accompanied by a marked shift toward younger age at onset (1). According to the European Society of Gynaecological Oncology/European Society for Radiotherapy and Oncology/European Society of Pathology (ESGO/ESTRO/ESP) 2025 guidelines, which integrate the International Federation of Gynecology and Obstetrics (FIGO) 2023 staging system, patients with EC without available molecular classification are stratified into four distinct risk groups: low, intermediate, high-intermediate, and high (2). Total hysterectomy with bilateral salpingo-oophorectomy is the standard surgical treatment for low-risk disease, whereas sentinel lymph node biopsy combined with ultrastaging can replace systematic lymphadenectomy and improve the accuracy of lymph node staging in early-stage disease. In cases of sentinel lymph node biopsy failure, side-specific lymphadenectomy is recommended for accurate nodal staging, particularly in patients at high-intermediate or high risk, and may also be considered for those at intermediate risk. As these guideline-concordant surgical decisions depend on accurate preoperative risk stratification, this study aimed to develop a binary classification model to preoperatively distinguish low-risk from non-low-risk disease.

Both the ESGO/ESTRO/ESP guidelines and the FIGO staging system rely on risk stratification criteria derived from postoperative surgical pathology findings; however, not all of these criteria can be accurately assessed preoperatively. In particular, lymphovascular space invasion (LVSI) and stage IA3 disease can only be reliably evaluated through postoperative pathological examination of hysterectomy specimens. This critical limitation directly impedes reliable preoperative risk stratification for patients with EC. Moreover, preoperative MRI assessment of myometrial invasion frequently does not correspond to postoperative pathological findings (3), and endometrial biopsy demonstrates variable concordance with final surgical pathology owing to factors such as insufficient specimen quality and interobserver variability (4, 5). Consequently, conventional preoperative evaluation alone cannot achieve the complete risk stratification defined by the guidelines, which may lead to overtreatment or undertreatment—particularly in decisions regarding lymphadenectomy for presumed low-risk patients (6).

Previous studies have demonstrated that routine clinical indicators are closely associated with EC risk stratification. Among these, age, body mass index (BMI), and CA125 have been identified as promising predictors for EC risk stratification (7–9). Tumor size also correlates significantly with EC risk stratification (9, 10). Building on this evidence, the present study aimed to develop a novel nomogram integrating these clinical and morphological predictors for preoperative EC risk stratification.

## 2 Methods

### 2.1 Patients and clinical data

This retrospective study was conducted in accordance with the Declaration of Helsinki and approved by the local institutional review board. Written informed consent was waived due to the retrospective design. A total of 286 consecutive patients with EC who underwent surgical treatment at a single tertiary referral center between March 2016 and June 2025 were initially screened. Inclusion criteria were as follows (1): adult patients (aged ≥ 18 years); (2) preoperative MRI at the center and total hysterectomy with final histopathological confirmation of EC. Exclusion criteria were as follows: (1) missing clinical, imaging, or endometrial biopsy data obtained within 1 month before surgery; (2) no available tumor size measurement on preoperative transvaginal ultrasound (TVUS); (3) suboptimal or incomplete MRI examinations; (4) synchronous pelvic malignancies. After applying the exclusion criteria, 35 patients were excluded, and a total of 251 patients were included in the final analysis (**Figure 1**).

**Figure 1:**
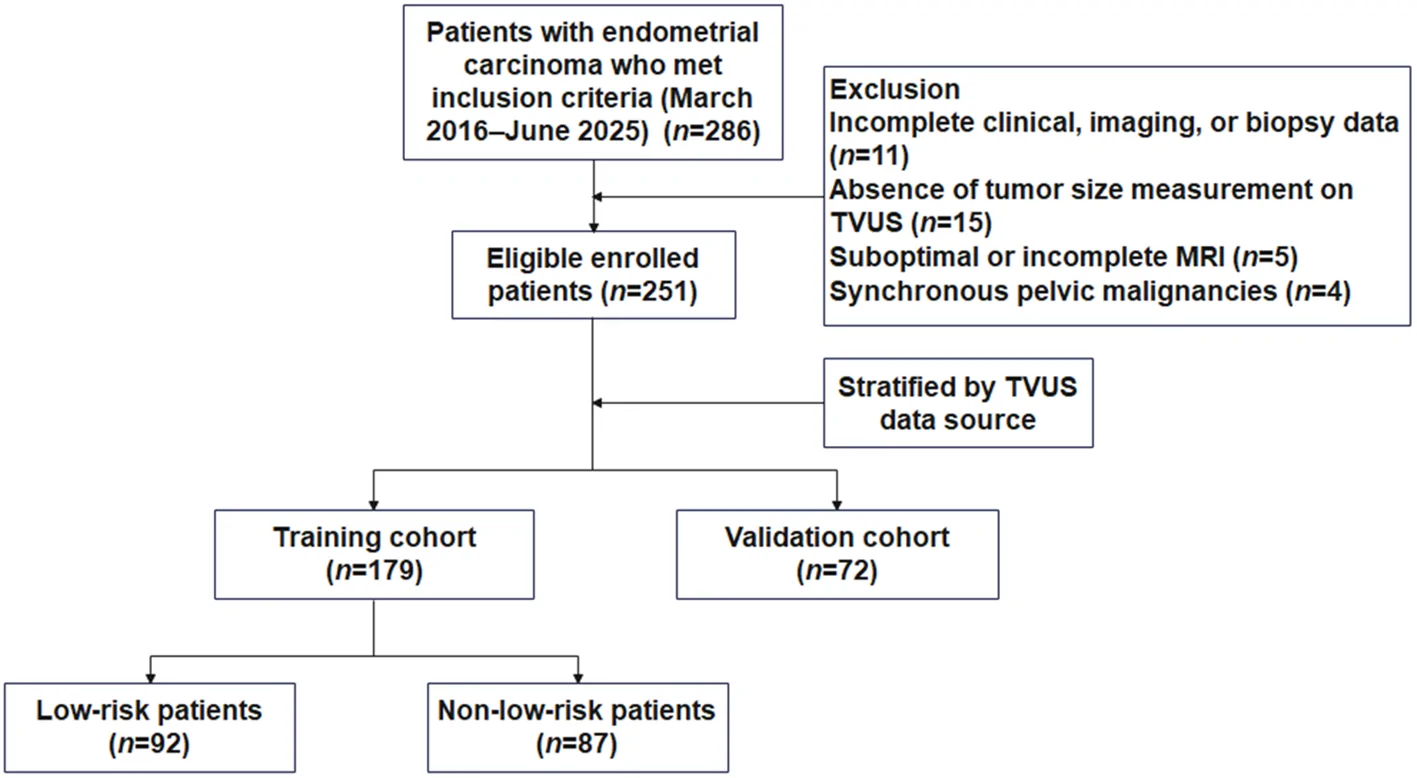
Flowchart of patient selection. TVUS, transvaginal ultrasound.

Clinical data collected included age, BMI, menopausal status, abnormal uterine bleeding, comorbidities (hypertension and diabetes mellitus), obstetric and gynecologic history (gravidity, parity, endometrial polyps, uterine leiomyoma, and adenomyosis), and preoperative serum CA125 and CA19–9 levels. Patients were stratified into two diagnostic pathways based on the order of preoperative examinations: Diagnostic Pathway 1 (endometrial biopsy performed prior to MRI) and Diagnostic Pathway 2 (MRI performed prior to endometrial biopsy). All data were extracted from the institutional electronic medical record system.

### 2.2 Preoperative imaging

All included patients underwent preoperative TVUS, with data extracted from the latest examination performed prior to total hysterectomy. TVUS data were retrieved from the institutional electronic medical record system or regional health archives. Patients were divided into two cohorts according to whether the preoperative TVUS was performed at our hospital or at external facilities: the training cohort (scanners predominantly GE Voluson E8, Mindray Nuewa R9G, and Mindray Resona 8S) and the validation cohort (scanners including GE, Mindray, Philips, Hitachi, and Toshiba). All TVUS scans from both cohorts were acquired following a standardized protocol, using transvaginal probes with a frequency range of 3–9 MHz. Examinations were performed by board-certified sonographers with extensive experience and specialized training in gynecological ultrasonography. Tumor dimensions were measured in three orthogonal planes on TVUS, and the maximum diameter across the three planes was recorded as tumor size. All measurements were performed strictly in accordance with the International Endometrial Tumor Analysis consensus (11), with sonographers blinded to final histopathological findings.

All patients underwent a standardized preoperative pelvic MRI examination. Before scanning, patients were instructed to fast for a minimum of 4 hours and maintain moderate bladder distention. MRI scans were acquired on a 1.5T Siemens Aera scanner with a 16-channel abdominal phased-array coil, with patients positioned supine for the entire examination. The standardized protocol included unenhanced and contrast-enhanced sequences; contrast-enhanced acquisitions were performed after intravenous administration of gadodiamide (0.2 mL/kg at an injection rate of 3 mL/s), with the scanning range covering from the iliac crest to the pubic symphysis. The imaging protocol was designed in accordance with European Society of Urogenital Radiology guidelines (12) and included the following sequences: (1) T2-weighted fast spin-echo sequences, acquired in sagittal, coronal oblique, and axial oblique planes (sagittal and axial oblique acquired with fat saturation), with the following parameters: repetition time/echo time, 3000–4500/86–121 ms; field of view, 300–400 mm; slice thickness, 4–5 mm; interslice gap, 0.4–0.5 mm; matrix, 256 × 192 to 336 × 224; (2) sagittal T1-weighted images (T1WI), acquired for all patients with repetition time/echo time, 500/8.1 ms; (3) diffusion-weighted images, acquired in the sagittal plane using a single-shot echo-planar imaging sequence with b-values of 0 and 800 s/mm^2^, with the following parameters: repetition time/echo time, 6900/74 ms; slice thickness, 4 mm; interslice gap, 0.4 mm; matrix, 128 × 192; field of view, 310 mm; number of excitations, 4; (4) unenhanced and contrast-enhanced fat-suppressed T1WI, acquired in sagittal, coronal oblique, and axial oblique planes using spectral attenuated inversion recovery, with images acquired at three sequential delay times (35 s, 90 s, and 150 s after intravenous contrast administration).

Two board-certified radiologists with 6 and 10 years of dedicated experience in pelvic MRI imaging, respectively, independently assessed MRI-based disease extent. Both radiologists were blinded to patients’ endometrial biopsy results, final histopathological findings, and TVUS data throughout the assessment period. Discrepancies were resolved by consensus with a third senior radiologist (with > 15 years of pelvic MRI experience), whose final assessment was adopted for all subsequent analyses.

For patients in Diagnostic Pathway 1, a radiologist with 10 years of dedicated pelvic MRI experience performed tumor size measurements. Measurements were conducted on equilibrium-phase (150 s after contrast administration) coronal oblique and axial oblique contrast-enhanced T1WI, with diffusion-weighted images and other sequences referenced for anatomical correlation. Tumor dimensions were measured in three orthogonal planes on MRI, and the maximum diameter across the three planes was recorded as tumor size.

### 2.3 Histopathology

The final histopathological findings served as the reference standard. This was based primarily on the comprehensive assessment of the hysterectomy specimen. In cases where no residual disease was identified postoperatively, the reference standard was established using a combination of the preoperative endometrial biopsy, confirmatory immunohistochemistry, and external pathological consultation when available. All histopathological assessments were performed in strict accordance with the WHO Classification of Female Genital Tumors, 5th edition and the current ESGO/ESTRO/ESP guidelines (2, 13).

Assessed pathological indicators included histological subtype and grade, depth of myometrial invasion, LVSI, cervical stromal invasion, and extrauterine invasion. A binary FIGO grading system was adopted to stratify EC into low-grade and high-grade subtypes: low-grade EC was defined as grade 1 or 2 endometrioid endometrial carcinoma (EEC), including mucinous adenocarcinoma; high-grade EC included grade 3 EEC and non-endometrioid EC, specifically serous carcinoma, clear cell carcinoma, carcinosarcoma, mixed histology, undifferentiated/dedifferentiated carcinoma, and other rare histological subtypes.

Low-risk EC was defined as low-grade EEC with myometrial invasion< 50%, negative or focal LVSI, and absence of cervical stromal invasion or extrauterine invasion. Non-low-risk EC was defined as cases with at least one of the following features: high-grade histology, deep myometrial invasion (≥ 50%), substantial LVSI, cervical stromal invasion, or extrauterine invasion. Substantial LVSI was defined as diffuse or multifocal involvement of lymphovascular spaces, or the presence of tumor cells in five or more lymphovascular spaces. All patients with EC and concurrent ovarian involvement were classified into the non-low-risk group, as the histological subtype and grade of ovarian lesions cannot be reliably determined preoperatively.

For endometrial biopsy specimens, low-grade EC was further subdivided into grade 1 and grade 2 EEC. Of note, mucinous adenocarcinoma was grouped with grade 1 EEC given its comparable clinical prognosis. Patients with an initial biopsy result of atypical hyperplasia/endometrioid intraepithelial neoplasia but a final finding of EC on postoperative histopathology were also included, with the biopsy result classified as grade 1 EEC. For cases with ambiguous subtype, grade, depth of myometrial invasion, LVSI, cervical stromal invasion, or extrauterine invasion, all pathological slides were re-reviewed by a board-certified gynecologic pathologist with more than 10 years of specialized experience in gynecologic oncology; the results of this review served as the final findings for all subsequent analyses.

### 2.4 Statistical analysis

All statistical analyses were performed using SPSS 27.0 and R 4.3.3, with a two-sided significance level set at *P<* 0.05. No missing data was present for any of the candidate predictors or the outcome variable. For baseline characteristics, continuous variables with non-normal distribution were expressed as median (interquartile range) and compared between groups using the Mann–Whitney *U* test; categorical variables were presented as number (percentage) and compared using the chi-square test or the linear-by-linear association test. Kappa values were used to evaluate agreement for dichotomized tumor size classification (cutoff: 2 cm). A nomogram prediction model was constructed using R, with least absolute shrinkage and selection operator (LASSO) regression for variable selection and logistic regression for final model construction. The following R packages were used: rms for logistic regression, nomogram generation, and bootstrap internal validation; pROC for receiver operating characteristic analysis; rmda for decision curve analysis (DCA); ggplot2 for visualization; and ResourceSelection for the Hosmer–Lemeshow test. Model performance was assessed in terms of discrimination, calibration, and clinical utility. Internal validation used bootstrap resampling to correct for optimism. The ability of the nomogram to improve risk reclassification beyond conventional assessment was assessed with continuous net reclassification improvement (NRI) and integrated discrimination improvement (IDI). The datasets and analysis code used in this study are available from the corresponding author upon reasonable request. This study was reported in accordance with the Transparent Reporting of a multivariable prediction model for Individual Prognosis or Diagnosis (TRIPOD) statement (14).

## 3 Results

### 3.1 Patient characteristics

Baseline characteristics and final histopathological findings of the 251 included patients were summarized in **Supplementary Table 1** and **Supplementary Table 2**. Agreement between TVUS and MRI for tumor size measurement was excellent (*κ* = 0.820, *P <* 0.001) (**Supplementary Table 3**). The study cohort was divided into training and validation cohorts, with no significant differences detected in baseline variables between groups (**Supplementary Table 4**). In the training cohort, univariate analysis revealed 10 variables with significant differences between low-risk and non-low-risk groups (**Table 1**). Following LASSO regression for variable selection (**Figure 2**), five candidate features were identified. Multivariable logistic regression subsequently confirmed BMI, tumor size (measured on TVUS), endometrial biopsy results, and MRI diagnosis as independent predictors, while age did not reach statistical significance and was therefore excluded from the final model (**Table 2**).

**Table 1:** Baseline characteristics by risk group in the training cohort.

Characteristics		Low-risk group (n = 92)	Non-low-risk group (n = 87)	*P* value
Age, years		53.0 (49.0, 63.8)	58.0 (52.0, 70.0)	0.004
BMI, kg/m^2^		25.1 (22.4, 28.6)	23.6 (22.1, 26.0)	0.018
Menopausal status	No	37 (40.2)	20 (23.0)	0.013
	Yes	55 (59.8)	67 (77.0)	
Abnormal uterine bleeding	No	25 (27.2)	14 (16.1)	0.073
	Yes	67 (72.8)	73 (83.9)	
History of hypertension	No	61 (66.3)	50 (57.5)	0.224
	Yes	31 (33.7)	37 (42.5)	
History of diabetes mellitus	No	78 (84.8)	79 (90.8)	0.220
	Yes	14 (15.2)	8 (9.2)	
Gravidity	0	9 (9.8)	4 (4.6)	0.336^*^
	1-2	41 (44.6)	40 (46.0)	
	≥ 3	42 (45.7)	43 (49.4)	
Parity	0	10 (10.9)	4 (4.6)	0.008^*^
	1-2	72 (78.3)	62 (71.3)	
	≥ 3	10 (10.9)	21 (24.1)	
History of endometrial polyps	No	82 (89.1)	81 (93.1)	0.352
	Yes	10 (10.9)	6 (6.9)	
History of uterine leiomyoma	No	58 (63.0)	69 (79.3)	0.017
	Yes	34 (37.0)	18 (20.7)	
History of adenomyosis	No	74 (80.4)	77 (88.5)	0.137
	Yes	18 (19.6)	10 (11.5)	
Serum CA125, kU/L	< 35	76 (82.6)	60 (69.0)	0.033
	≥ 35	16 (17.4)	27 (31.0)	
Serum CA19-9, kU/L	< 35	82 (89.1)	67 (77.0)	0.030
	≥ 35	10 (10.9)	20 (23.0)	
Tumor size, cm	< 2	37 (40.2)	18 (20.7)	0.005
	≥ 2	55 (59.8)	69 (79.3)	
Endometrial biopsy	G1	61 (66.3)	20 (23.0)	< 0.001^*^
	G2	17 (18.5)	19 (21.8)	
	G3/NEEC	14 (15.2)	48 (55.2)	
MRI diagnosis	MI< 1/2	82 (89.1)	35 (40.2)	< 0.001
	MI ≥ 1/2, CI, or EI	10 (10.9)	52 (59.8)	

BMI, body mass index; G, grade; NEEC, non-endometrioid endometrial carcinoma; MI, myometrial invasion; CI, cervical invasion; EI, extrauterine invasion.

Low-risk and non-low-risk endometrial carcinoma were defined according to the ESGO/ESTRO/ESP 2025 guidelines. Tumor size was measured by transvaginal ultrasound; G1, G2, and G3 are histological grades of endometrioid endometrial carcinoma.

Data in bold indicates *p <* 0.05.

*Linear-by-linear association test.

**Figure 2:**
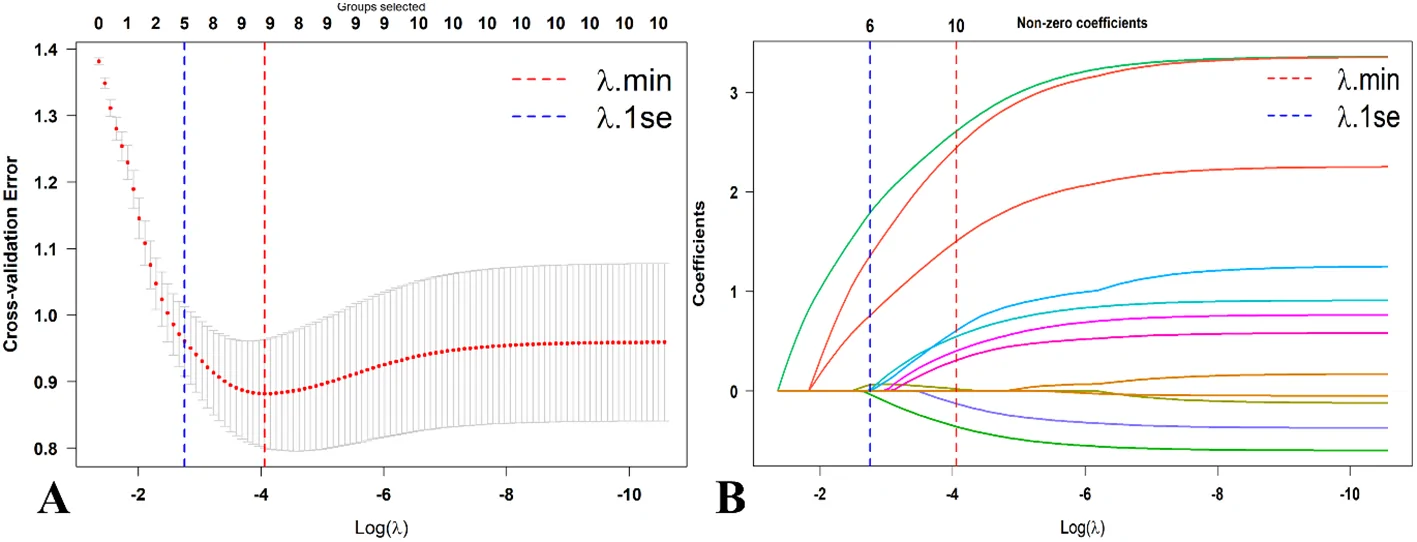
Feature selection using Group Least Absolute Shrinkage and Selection Operator regression with tenfold cross-validation. **(A)** Cross-validation error curve as a function of log(λ). The optimal λ (left dashed line) was selected based on the one-standard-error rule (λ.1se). **(B)** Coefficient profiles plotted against log(λ). Ten candidate predictors (yielding 12 coefficients after expanding two categorical features with two dummy variables each) were entered; five predictors (including two dummy variables from one categorical feature) were retained at this optimal λ for further analysis.

**Table 2:** Univariate and multivariable logistic regression analyses of factors associated with non-low-risk endometrial carcinoma in the training cohort.

Variables	Univariate analysis			Multivariable analysis		
	*OR*	95% *CI*	*P* value	*OR*	95% *CI*	*P value*
Age, years	1.042	1.013-1.072	0.005	–	–	–
BMI, kg/m^2^	0.923	0.860-0.990	0.026	0.862	0.773-0.961	0.007
Tumor size (≥ 2 cm)	2.579	1.326-5.016	0.005	2.880	1.108-7.482	0.030
G1	reference	–	–	–	–	–
G2	3.409	1.492-7.791	0.004	8.625	2.546-29.215	< 0.001
G3/NEEC	10.457	4.790-22.828	< 0.001	24.832	7.899-78.067	< 0.001
MRI features	12.183	5.563-26.683	< 0.001	29.101	9.363-90.448	< 0.001

*OR*, odds ratio; BMI, body mass index; G, grade; NEEC, non-endometrioid endometrial carcinoma.

Tumor size was measured by transvaginal ultrasound; G1, G2, and G3 are histological grades of endometrioid endometrial carcinoma, and both G1–G3 and NEEC were determined by endometrial biopsy; MRI features included myometrial invasion ≥ 1/2, cervical invasion, or extrauterine invasion.

Data in bold indicates *p<* 0.05.

### 3.2 Development and validation of the nomogram

Based on the four independent predictors identified, a nomogram model was constructed to predict the risk of non-low-risk disease (**Figure 3**), with an optimal cut-off value of 138 points. The model is expressed as:

**Figure 3:**
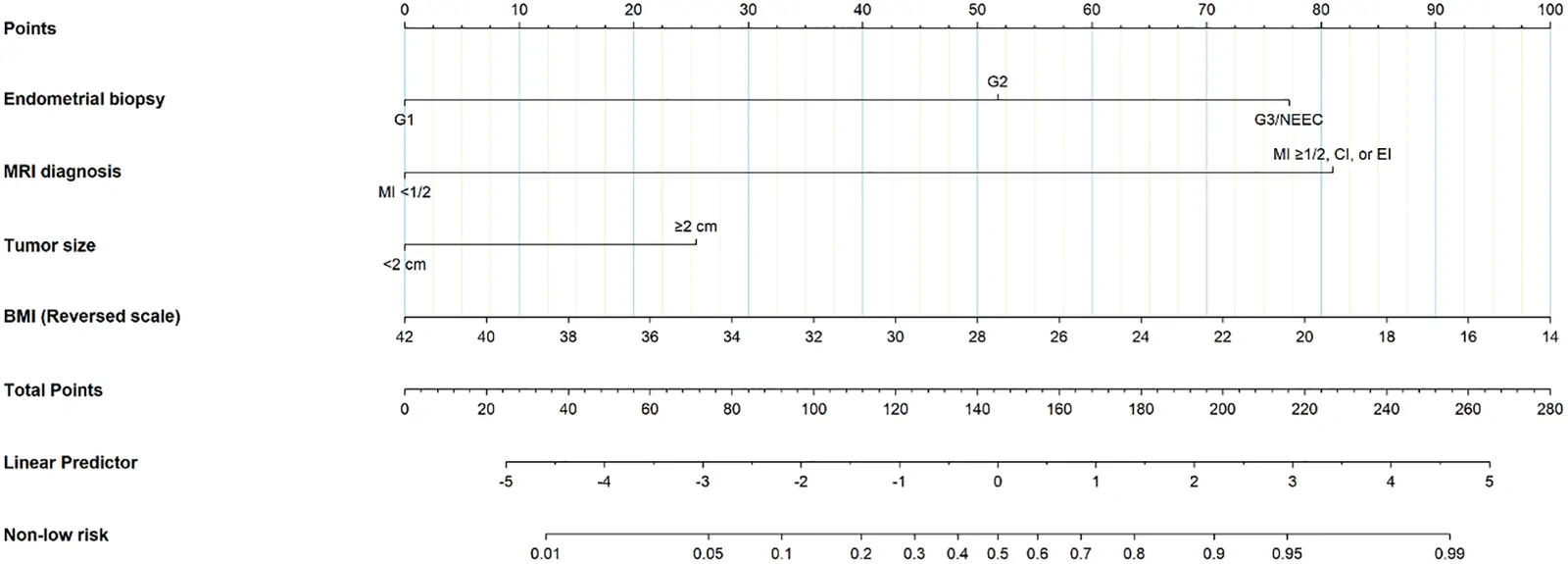
Nomogram for preoperative risk stratification of endometrial carcinoma. The optimal Youden-based cutoff is a total score of 138 (probability 0.4294; linear predictor -0.2844). The reversed BMI axis reflects its inverse association with risk. G, grade; NEEC, non-endometrioid endometrial carcinoma; MI, myometrial invasion; CI, cervical invasion; EI, extrauterine invasion; BMI, body mass index. G1, G2, and G3 are histological grades of endometrioid endometrial carcinoma; Tumor size was measured by transvaginal ultrasound.


$$\text{logit}\left(P\right)=0.209+2.155\times \left(\text{grade }2\text{ EEC}\right)+3.212\times \left(\text{grade }3\text{ EEC}/\text{non-endometrioid EC}\right)+3.371\times \left(\text{deep myometrial}/\text{cervical involvement}/\text{extrauterine spread on MRI}\right)+1.058\times \left(\text{tumor size}\geq 2\text{ cm}\right)-0.149\times \text{BMI}\left(\text{kg}/{\text{m}}^{2}\right).$$

Where *P* denotes the predicted probability of non-low-risk EC. Histological grade was treated as a categorical variable with grade 1 as the reference, and entered the model as two indicator variables. MRI findings and tumor size were coded as binary variables (1 if present, 0 otherwise). BMI was included as a continuous variable, with the coefficient representing the change in log-odds per 1 kg/m^2^ increment. The optimal probability threshold, determined by the Youden index, was 0.4294, which corresponded to a total nomogram score of 138 points (rounded from 138.1) and a predicted probability of 42.8%.

The model demonstrated high discrimination, with an area under the curve (AUC) of 0.902 (95% *CI*: 0.857–0.948) in the training cohort and 0.866 (95% *CI*: 0.773–0.959) in the validation cohort (**Table 3**; **Figure 4**). Bootstrap resampling (1,000 resamples) of the training cohort yielded an optimism-corrected AUC of 0.892 (95% *CI*: 0.885–0.902), along with corrected calibration intercept (0.008), calibration slope (1.093), and Brier score (0.128); the Hosmer–Lemeshow test also showed good calibration (*χ*^2^ = 11.895, *P* = 0.156).

**Table 3:** Discriminatory performance of the nomogram versus biopsy combined with MRI for preoperative risk stratification.

Models		AUC (95% *CI*)	ACC (95% *CI*)	SENS (95% *CI*)	SPEC (95% *CI*)	PPV (95% *CI*)	NPV (95% *CI*)	SE
Training Cohort (n = 179)	Biopsy + MRI	0.812 (0.746-0.878)	0.810 (0.745-0.865)	0.874 (0.785-0.935)	0.750 (0.649-0.834)	0.768 (0.672-0.847)	0.863 (0.767-0.929)	0.034
	Nomogram	0.902 (0.857-0.948)	0.849 (0.788-0.898)	0.908 (0.827-0.959)	0.793 (0.696-0.871)	0.806 (0.714-0.879)	0.901 (0.815-0.956)	0.023
Validation Cohort (n = 72)	Biopsy + MRI	0.797 (0.687-0.907)	0.806 (0.695-0.889)	0.697 (0.513-0.844)	0.897 (0.758-0.971)	0.852 (0.663-0.958)	0.778 (0.629-0.888)	0.056
	Nomogram	0.866 (0.773-0.959)	0.833 (0.727-0.911)	0.788 (0.611-0.910)	0.872 (0.726-0.957)	0.839 (0.663-0.945)	0.829 (0.679-0.928)	0.047

AUC, area under the curve; ACC, accuracy; SENS, sensitivity; SPEC, specificity; PPV, positive predictive value; NPV, negative predictive value; SE, standard error.

**Figure 4:**
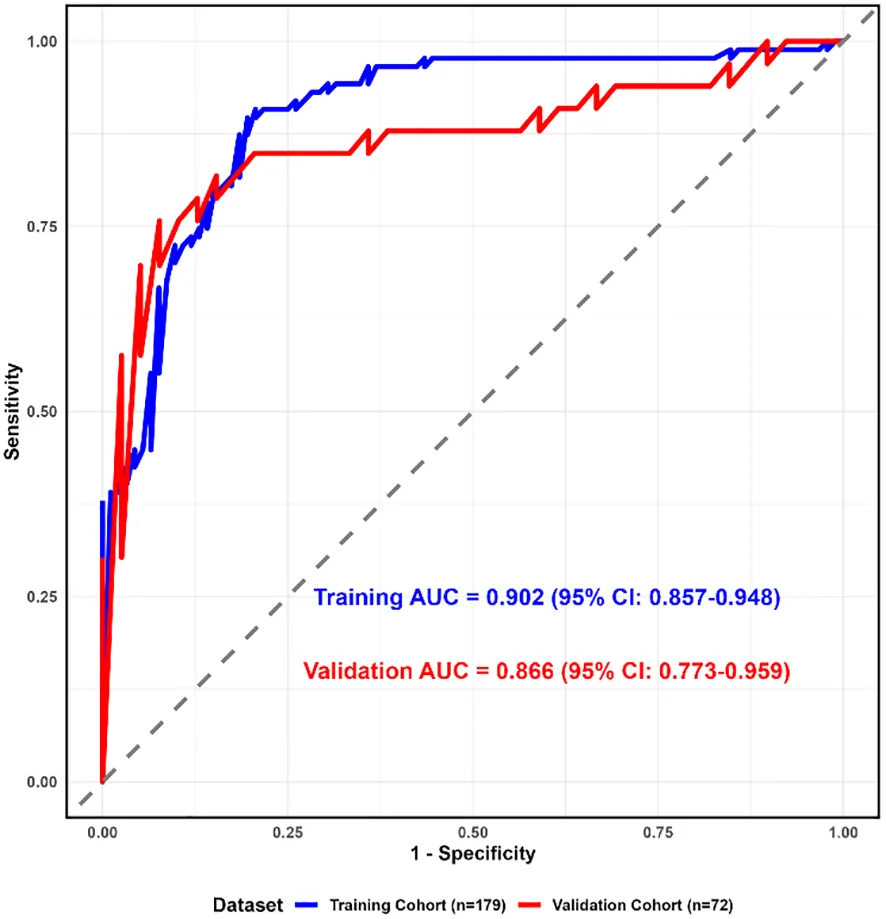
Receiver operating characteristic curves of the nomogram in the training and validation cohorts.

In the training cohort, the nomogram achieved a significantly higher AUC than conventional assessment (*Z* = 3.982, *P <* 0.001). Cross-cohort stability was supported by the Hanley–McNeil test, which revealed no significant difference in AUC between the training and validation cohorts (*Z* = 0.688, *P* = 0.491). In the validation cohort, the nomogram showed a non-significant AUC increase over conventional assessment (*Δ*AUC = 0.069; DeLong test, *Z* = 1.845, *P* = 0.065). Calibration was satisfactory (intercept, 0.417 [SE, 0.349]; slope, 0.810 [SE, 0.189]; Brier score, 0.142; **Figure 5**). DCA showed favorable net benefit over conventional assessment at threshold probabilities of 0.101–0.113, 0.158–0.916, and 0.944–0.969 (**Figure 6**). Furthermore, the nomogram provided significant improvements in risk reclassification and discrimination (continuous NRI = 1.189, 95% *CI*: 0.193–2.185, *P* = 0.019; IDI = 0.126, 95% *CI*: 0.027–0.221, *P* = 0.013).

**Figure 5:**
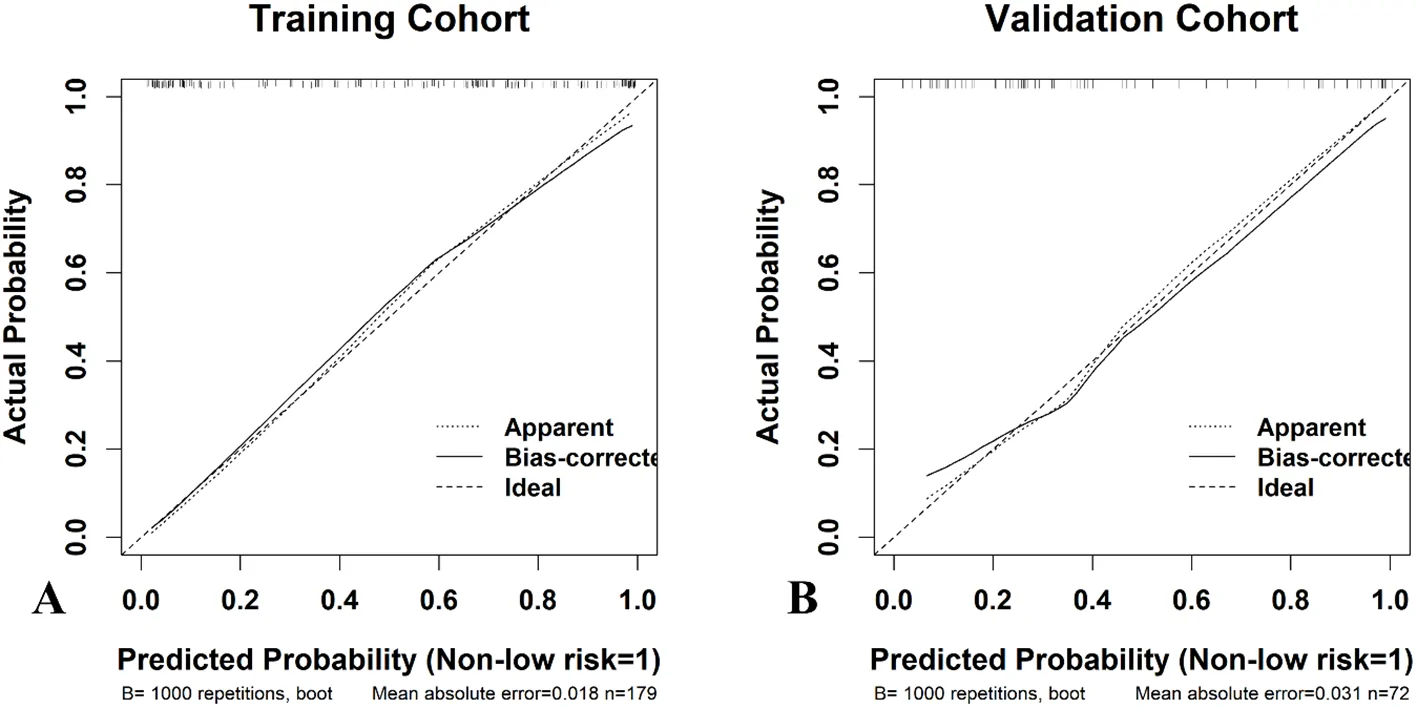
Calibration curves. **(A)** Training cohort: intercept = 0.000 (SE 0.212), slope = 1.000 (SE 0.147), Brier score = 0.122. **(B)** Validation cohort: intercept = 0.417 (SE 0.349), slope = 0.810 (SE 0.189), Brier score = 0.142.

**Figure 6:**
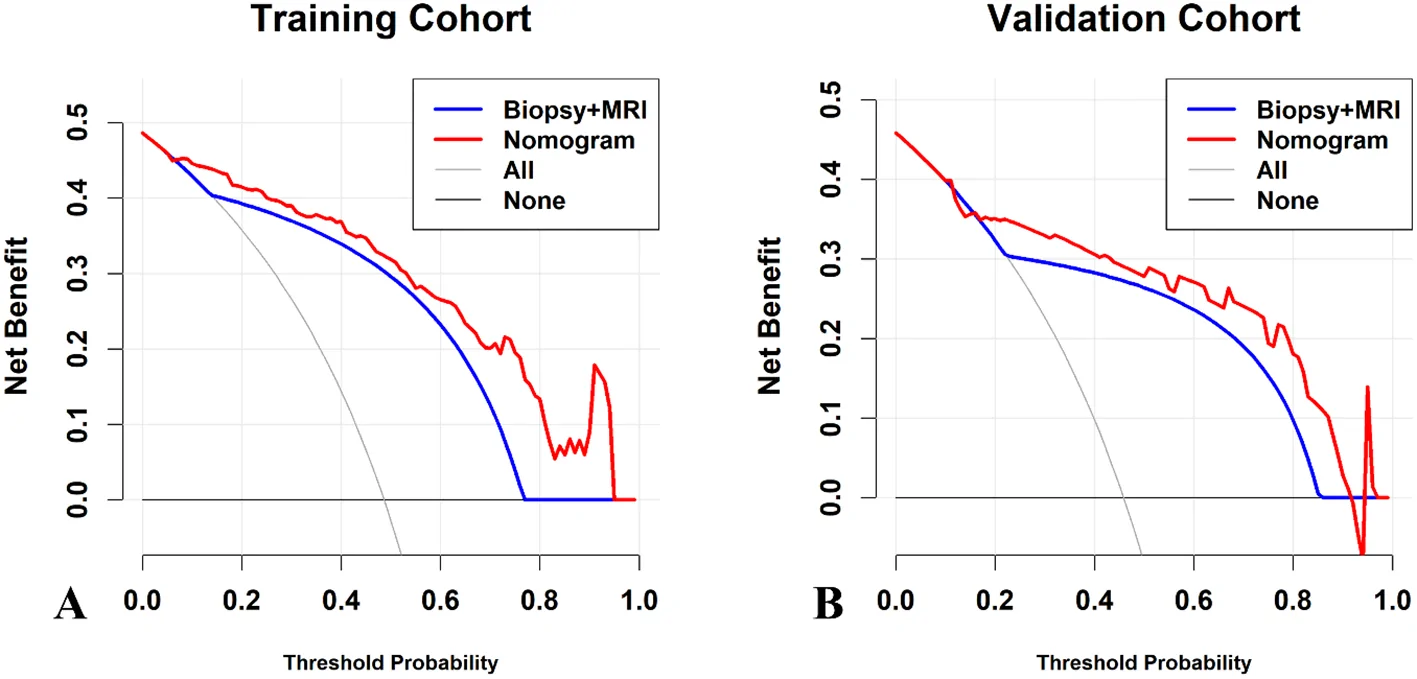
Decision curve analysis of the nomogram (red) versus conventional assessment combining biopsy with MRI (blue). In the training cohort, the nomogram demonstrated greater net benefit at threshold probabilities of 0.041–0.051 and 0.065–0.949, was equivalent at 0–0.041 and 0.949–1.000, and showed slightly lower net benefit only at 0.051–0.065. In the validation cohort, greater net benefit was observed at 0.101–0.113, 0.158–0.916, and 0.944–0.969, with equivalence at 0–0.101 and 0.969–1.000, and lower net benefit at 0.113–0.158 and 0.916–0.944.

### 3.3 Sensitivity analysis

A total of 286 patients were screened, of whom 35 (12.2%) were excluded according to the exclusion criteria (**Figure 1**). In the overall cohort, six patients (2.4%) had no residual disease at hysterectomy and their final histopathological findings were established postoperatively using immunohistochemistry and external consultation; a separate patient was postoperatively found to have stage IA3 disease and was misclassified as non-low-risk by both the nomogram (total score, 147; predicted probability, 51.6%) and conventional assessment. Of the 28 patients with substantial LVSI, 3 (10.7%) were not identified as non-low-risk by the nomogram and none were detected by conventional assessment, including two in the training cohort (total scores, 127 and 132; predicted probabilities, 32.1% and 36.8%) and one in the validation cohort (total score, 130; predicted probability, 34.9%). In the validation cohort, conventional assessment missed 10 non-low-risk patients; the nomogram correctly reclassified three of these, reducing the false-negative rate from 30.3% to 21.2%. Kappa values for agreement between preoperative assessment and final histopathology are presented for the overall, training, and validation cohorts in **Supplementary Figures 1–3**, respectively.

## 4 Discussion

In this study, we developed and validated a nomogram integrating clinical, pathological, and radiological indicators to preoperatively differentiate low-risk from non-low-risk patients. The model demonstrated robust discrimination in the training cohort and satisfactory calibration in the validation cohort. Notably, while the improvement in AUC over conventional assessment (endometrial biopsy combined with MRI) did not reach statistical significance in the validation cohort, the nomogram showed favorable net benefit on DCA and improved risk reclassification. Of note, the model showed stable performance when TVUS data from external facilities were used in the validation cohort. Unlike previously published models that rely on specialized testing or dedicated equipment, our model is based solely on routinely available examinations and common clinical variables, which support its potential role as a pragmatic complementary decision-support tool.

In our cohort, conventional assessment alone offered limited discrimination, with sensitivity, specificity, and accuracy comparable to those reported by Nakai et al. (15). In that study, the addition of frozen section analysis improved sensitivity only marginally (78.3% to 81.2%, *P* = 0.16). Radiomics-based models have since achieved higher preoperative accuracy, with previous studies reporting AUCs of 0.874–0.929 for imaging features alone and 0.869–0.949 when combined with clinical variables (16–18); our nomogram’s AUC of 0.902 (95% *CI*: 0.857–0.948) falls within this range. However, the generalizability of radiomics approaches is constrained by the lack of standardized imaging protocols and variability in feature extraction and selection pipelines. By relying solely on routinely available variables, our model may serve as a pragmatic complementary tool, easily implementable option for preoperative risk stratification, particularly when advanced imaging post-processing is unavailable or its quality is suboptimal.

LASSO regression was applied to ten candidate variables for feature selection, and the retained variables were then assessed by multivariable logistic regression to confirm their independent effects; age was excluded because it was not significant. Among the remaining predictors, BMI was confirmed as an independent predictor (*P* = 0.007), consistent with prior studies (8, 19). The observed inverse association between BMI and non-low-risk disease is biologically plausible: aggressive tumor types tend to concentrate in non-obese patients, leading to an uneven distribution of histological risk across BMI categories (20). Tumor size was also confirmed as an independent predictor (*OR* = 2.880, *P* = 0.030), reinforcing its role in preoperative EC risk stratification (21). For women with suspected EC, initial evaluation routinely includes TVUS, but the sequence of subsequent MRI and endometrial biopsy varies across clinical settings (22). Because post-biopsy changes can obscure tumor margins on MRI and the timing of MRI relative to biopsy varied in our cohort (45.8% had MRI after biopsy), we measured tumor size uniformly by TVUS to avoid systematic bias. In the subset with both examinations before biopsy, TVUS and MRI showed excellent agreement (*κ* = 0.820, *P* < 0.001), consistent with previous studies (23, 24). This approach ensures that tumor size remains a standardized predictor independent of MRI timing and modality, consistent with the model’s complementary design.

Notably, this reclassification improvement translated into a clinically meaningful reduction in the false-negative rate from 30.3% to 21.2% in the validation cohort. The nomogram can serve as an objective reference to aid decision-making when conventional assessment alone offers insufficient guidance for surgery. Consider the patient for whom sentinel lymph node mapping is not an option, or in whom it is attempted but fails, with failure rates of 4%–22% reported in the literature (25). Likewise, for patients whose comorbidities limit their ability to undergo prolonged surgery and who face a heightened risk of complications from comprehensive lymphadenectomy, including lymphatic fistula and lymphedema (26), a more precise risk estimate becomes essential. Nonetheless, these findings derive from a single-center retrospective cohort with a limited number of events in the validation cohort; the observed reclassification benefit and its impact on surgical decision-making require confirmation in prospective multicenter studies before clinical implementation can be recommended. False-negative predictions remain possible, and the nomogram should complement, not replace, multidisciplinary clinical judgment.

The key challenges for clinical translation of EC risk stratification models lie in defining the applicable population, managing extreme cases, and addressing the variability of predictor acquisition across institutions. This nomogram is intended for all newly diagnosed EC patients, and the high inclusion rate (87.8%) suggests that the enrolled cohort adequately represents the target population, with minimal selection bias. Nevertheless, substantial LVSI and stage IA3 are generally not assessable before surgery, representing a shared limitation of current preoperative evaluation rather than a specific shortcoming of the nomogram. For patients with a high predicted risk approaching the non-low-risk threshold, clinicians may consider additional preoperative assessment strategies to further refine the risk estimate. Regarding the variability in predictor acquisition, prior studies have attempted to ensure consistent nomogram performance across heterogeneous scanning parameters by stratifying patients according to MR field strength and acquisition protocols (10, 16). As noted earlier, the model’s discriminative performance remained stable irrespective of the source of TVUS examination, supporting its applicability across different clinical settings.

This study has several limitations. First, the nomogram was developed without molecular classification, which is increasingly regarded as an important component of EC risk stratification (27). For patients with available preoperative molecular profiling, this information can supplement the nomogram-based assessment to refine risk stratification. As molecular profiling becomes more routinely accessible, the nomogram could provide a clinical-imaging framework into which molecular subtypes are incorporated, enabling a more comprehensive preoperative stratification system. Second, tumor size is a validated morphological indicator for EC risk stratification (9, 10); future work could explore optimal strategies for integrating quantitative morphological features into predictive models, potentially further improving reclassification performance. In summary, this nomogram provides a pragmatic, complementary tool for preoperative risk stratification in EC, with its clinical utility to be established through prospective multicenter validation.

## 5 Conclusion

By integrating BMI and tumor size with conventional assessment, this nomogram offers a pragmatic tool for distinguishing low-risk from non-low-risk EC before surgery. The model showed robust discrimination in the training cohort and calibrated acceptably in the validation cohort. Although the improvement in AUC over conventional assessment did not reach statistical significance, the nomogram achieved favorable net benefit on DCA and meaningful gains in risk reclassification. These findings support its potential role as a complementary decision aid in scenarios where conventional evaluation alone is insufficient. As this study was retrospective and conducted at a single center, the model’s clinical utility and its influence on surgical decision-making await validation in prospective multicenter studies.

## Data Availability

The datasets and corresponding analysis code for this study can be obtained from the corresponding author upon reasonable request. Access is subject to the author’s approval, and a formal data-sharing agreement will be required. The code is additionally deposited in the Science Data Bank repository (https://doi.org/10.57760/sciencedb.34323).

## Glossary

EC: Endometrial Carcinoma
AUC: Area Under The Curve
DCA: Decision Curve Analysis
NRI: Net Reclassification Improvement
IDI: Integrated Discrimination Improvement
ESGO/ESTRO/ESP: European Society of Gynaecological Oncology/European Society for Radiotherapy and Oncology/European Society of Pathology
FIGO: International Federation of Gynecology and Obstetrics
LVSI: lymphovascular Space Invasion
BMI: Body Mass Index
TVUS: Transvaginal Ultrasound
T1WI: T1-Weighted Images
EEC: Endometrioid Endometrial Carcinoma
LASSO: Least Absolute Shrinkage and Selection Operator.
