# Supplementary material for: Preoperative risk stratification in endometrial carcinoma: a pragmatic, complementary nomogram using readily available clinical variables
Source: Front Oncol. 2026 Sep 22;16:1894175. doi: 10.3389/fonc.2026.1894175 (PMC13638399; doi:10.3389/fonc.2026.1894175)
Supplement: Supplementary file 1 [file Table1.docx]

**Supplementary Table 1.** Baseline characteristics and comparison of diagnostic pathways in patients with endometrial carcinoma

| **Characteristics** | | **Total**  **(n=251)** | **Diagnostic Pathway 1**  **(*n*=136)** | **Diagnostic Pathway 2**  **(*n*=115)** | ***P* value** |
| --- | --- | --- | --- | --- | --- |
| Risk stratification | Low-risk | 131(52.2) | 67(49.3) | 64(55.7) | 0.313 |
|  | Non-low-risk | 120(47.8) | 69(50.7) | 51(44.3) |  |
| Age, years |  | 55.0(50.0, 67.0) | 60.5(52.0, 69.8) | 53(48.0, 60.0) | **< 0.001** |
| BMI, kg/m^2^ |  | 24.4(22.2, 27.4) | 24.8(22.2, 28.0) | 23.9(22.2, 27.0) | 0.152 |
| Menopausal status | No | 80(31.9) | 36(26.5) | 44(38.3) | **0.046** |
|  | Yes | 171(68.1) | 100(73.5) | 71(61.7) |  |
| Abnormal uterine bleeding | No | 50(19.9) | 23(16.9) | 27(23.5) | 0.194 |
|  | Yes | 201(80.1) | 113(83.1) | 88(76.5) |  |
| History of hypertension | No | 155(61.8) | 79(58.1) | 76(66.1) | 0.194 |
|  | Yes | 96(38.2) | 57(41.9) | 39(33.9) |  |
| History of diabetes mellitus | No | 217(86.5) | 113(83.1) | 104(90.4) | 0.090 |
|  | Yes | 34(13.5) | 23(16.9) | 11(9.6) |  |
| Gravidity | 0 | 17(6.8) | 11(8.1) | 6(5.2) | 0.217^*^ |
|  | 1-2 | 110(43.8) | 50(36.8) | 60(52.2) |  |
|  | ≥3 | 124(49.4) | 75(55.1) | 49(42.6) |  |
| Parity | 0 | 18(7.2) | 11(8.1) | 7(6.1) | 0.309^*^ |
|  | 1-2 | 189(75.3) | 96(70.6) | 93(80.9) |  |
|  | ≥3 | 44(17.5) | 29(21.3) | 15(13.0) |  |
| History of endometrial polyps | No | 226(90.0) | 120(88.2) | 106(92.2) | 0.299 |
|  | Yes | 25(10.0) | 16(11.8) | 9(7.8) |  |
| History of uterine leiomyoma | No | 169(67.3) | 91(66.9) | 78(67.8) | 0.878 |
|  | Yes | 82(32.7) | 45(33.1) | 37(32.2) |  |
| History of adenomyosis | No | 206(82.1) | 114(83.8) | 92(80.0) | 0.431 |
|  | Yes | 45(17.9) | 22(16.2) | 23(20.0) |  |
| Serum CA125, kU/L | <35 | 196(78.1) | 105(77.2) | 91(79.1) | 0.713 |
|  | ≥35 | 55(21.9) | 31(22.8) | 24(20.9) |  |
| Serum CA19-9, kU/L | <35 | 205(81.7) | 107(78.7) | 98(85.2) | 0.182 |
|  | ≥35 | 46(18.3) | 29(21.3) | 17(14.8) |  |
| Tumor size, cm | <2 | 76(30.3) | 28(20.6) | 48(41.7) | **< 0.001** |
|  | ≥2 | 175(69.7) | 108(79.4) | 67(58.3) |  |
| Endometrial biopsy | G1 | 119(47.4) | 58(42.6) | 61(53.0) | 0.063^*^ |
|  | G2 | 55(21.9) | 30(22.1) | 25(21.7) |  |
|  | G3/NEEC | 77(30.7) | 48(35.3) | 29(25.2) |  |
| MRI diagnosis | MI <1/2 | 170(67.7) | 89(65.4) | 81(70.4) | 0.399 |
|  | MI ≥1/2, CI, or EI | 81(32.3) | 47(34.6) | 34(29.6) |  |

Abbreviations: BMI, body mass index; G, grade; NEEC, non-endometrioid endometrial carcinoma;MI, myometrial invasion; CI, cervical invasion; EI, extrauterine invasion.

**Note:** Low-risk and non-low-risk endometrial carcinoma were defined according to the ESGO/ESTRO/ESP 2025 guidelines; Tumor size was measured by transvaginal ultrasound; G1, G2, and G3 are histological grades of endometrioid endometrial carcinoma.

Data in bold indicate *p*<0.05.

*Linear-by-linear association test.

**Supplementary Table 2.** Final postoperative histopathological findings in 251 patients with endometrial carcinoma

| **Histological characteristics** | | **Number (%)** |
| --- | --- | --- |
| Histological type | Endometrioid | 209(83.3) |
|  | Serous | 15(6.0) |
|  | Clear cell | 8(3.2) |
|  | Carcinosarcoma | 8(3.2) |
|  | Mixed histology | 5(2.0) |
|  | Undifferentiated/dedifferentiated | 3(1.2) |
|  | Others | 3(1.2) |
| Histological grade | G1 | 94(45.0) |
|  | G2 | 80(38.3) |
|  | G3 | 35(16.7) |
| MI | <1/2 | 202(80.5) |
|  | ≥1/2 | 49(19.5) |
| Lymphovascular  space invasion | negative or focal | 223(88.8) |
|  | substantial | 28(11.2) |
| CI | Absent | 225(89.6) |
|  | Present | 26(10.4) |
| EI | Absent | 209(83.3) |
|  | Present | 42(16.7) |
| FIGO 2023 Stage | IA1 | 50(19.9) |
|  | IA2 | 81(32.3) |
|  | IA3 | 1(0.4) |
|  | IB | 15(6.0) |
|  | IC | 11(4.4) |
|  | IIA | 7(2.8) |
|  | IIB | 7(2.8) |
|  | IIC | 38(15.1) |
|  | III/IV | 41(16.3) |

Abbreviations: G, grade; MI, myometrial invasion; CI, cervical invasion; EI, extrauterine invasion; FIGO, International Federation of Gynecology and Obstetrics.

**Note:** Final histopathological findings were established through comprehensive assessment of hysterectomy specimens, supplemented by preoperative endometrial biopsy specimens (in cases without residual disease on postoperative evaluation), immunohistochemical staining, and external pathological consultation reports were available; Histological grades are applicable only to endometrioid endometrial carcinoma; FIGO 2023 Stage was assigned based on the final histopathological findings only; molecular classification data were not integrated into the staging assessment presented in this table.

**Supplementary Table 3.** Agreement between transvaginal ultrasound and MRI for tumor size quantification in patients with endometrial carcinoma

|  | | **TVUS** | | **Total** |
| --- | --- | --- | --- | --- |
|  |  | **<2** | **≥2** |  |
| **MRI** | **<2** | 24 | 4 | 28 |
|  | **≥2** | 4 | 104 | 108 |
|  | **Total** | 28 | 108 | 136 |
| **Kappa value** | | 0.820 | | |
| ***P* value** | | *< 0.001* | | |

Abbreviations: TVUS, transvaginal ultrasound.

Note: Analysis was restricted to patients assigned to Diagnostic Pathway 1. Tumor size was defined as the maximum diameter. Agreement between TVUS and MRI measurements was assessed using Cohen's kappa (κ).

**Supplementary Table 4.** Baseline characteristics of the Training and Validation Cohorts

| **Characteristics** | | **Training Cohort**  **(*n*=179)** | **Validation Cohort**  **(*n*=72)** | ***P* value** |
| --- | --- | --- | --- | --- |
| Age, years |  | 55.0(50.0, 67.0) | 56.5(51.0, 67.5) | 0.849 |
| BMI, kg/m^2^ |  | 24.3(22.2, 27.4) | 24.6(22.1, 27.4) | 0.999 |
| Menopausal status | No | 57(31.8) | 23(31.9) | 0.988 |
|  | Yes | 122(68.2) | 49(68.1) |  |
| Abnormal uterine bleeding | No | 39(21.8) | 11(15.3) | 0.243 |
|  | Yes | 140(78.2) | 61(84.7) |  |
| History of hypertension | No | 111(62.0) | 44(61.1) | 0.894 |
|  | Yes | 68(38.0) | 28(38.9) |  |
| History of diabetes mellitus | No | 157(87.7) | 60(83.3) | 0.359 |
|  | Yes | 22(12.3) | 12(16.7) |  |
| Gravidity | 0 | 13(7.3) | 4(5.6) | 0.331^*^ |
|  | 1-2 | 81(45.3) | 29(40.3) |  |
|  | ≥ 3 | 85(47.5) | 39(54.2) |  |
| Parity | 0 | 14(7.8) | 4(5.6) | 0.659^*^ |
|  | 1-2 | 134(74.9) | 55(76.4) |  |
|  | ≥3 | 31(17.3) | 13(18.1) |  |
| History of endometrial polyps | No | 163(91.1) | 63(87.5) | 0.394 |
|  | Yes | 16(8.9) | 9(12.5) |  |
| History of uterine leiomyoma | No | 127(70.9) | 42(58.3) | 0.054 |
|  | Yes | 52(29.1) | 30(41.7) |  |
| History of adenomyosis | No | 151(84.4) | 55(76.4) | 0.137 |
|  | Yes | 28(15.6) | 17(23.6) |  |
| Serum CA125, kU/L | <35 | 136(76.0) | 60(83.3) | 0.203 |
|  | ≥35 | 43(24.0) | 12(16.7) |  |
| Serum CA19-9, kU/L | <35 | 149(83.2) | 56(77.8) | 0.312 |
|  | ≥35 | 30(16.8) | 16(22.2) |  |
| Tumor size, cm | <2 | 55(30.7) | 21(29.2) | 0.808 |
|  | ≥2 | 124(69.3) | 51(70.8) |  |
| Endometrial biopsy | G1 | 81(45.3) | 38(52.8) | 0.079^*^ |
|  | G2 | 36(20.1) | 19(26.4) |  |
|  | G3/NEEC | 62(34.6) | 15(20.8) |  |
| MRI diagnosis | MI <1/2 | 117(65.4) | 53(73.6) | 0.206 |
|  | MI ≥1/2, CI, or EI | 62(34.6) | 19(26.4) |  |

Abbreviations: BMI, body mass index; G, grade; NEEC, non-endometrioid endometrial carcinoma;MI, myometrial invasion; CI, cervical invasion; EI, extrauterine invasion.

Note: Tumor size was measured by transvaginal ultrasound; G1, G2, and G3 are histological grades of endometrioid endometrial carcinoma.

*Linear-by-linear association test.

**Supplementary Figures**

**Supplementary Figure 1.** Agreement between preoperative assessment and final histopathology in the overall cohort. (A) MRI assessment versus postoperative invasion reference. (B) Endometrial biopsy versus final histologic reference. (C) Endometrial biopsy combined with MRI versus final risk stratification. (D) Nomogram versus final risk stratification.

Abbreviations: MI, myometrial invasion; CI, cervical invasion; EI, extrauterine invasion; G, grade; NEEC, non-endometrioid endometrial carcinoma.

Note: G1, G2, and G3 are histological grades of endometrioid endometrial carcinoma.

**Supplementary Figure 2.** Agreement between preoperative assessment and final histopathology in the training cohort. (A) MRI assessment versus postoperative invasion reference. (B) Endometrial biopsy versus final histologic reference. (C) Endometrial biopsy combined with MRI versus final risk stratification. (D) Nomogram versus final risk stratification.

Abbreviations: MI, myometrial invasion; CI, cervical invasion; EI, extrauterine invasion; G, grade; NEEC, non-endometrioid endometrial carcinoma.

Note: G1, G2, and G3 are histological grades of endometrioid endometrial carcinoma.

**Supplementary Figure 3.** Agreement between preoperative assessment and final histopathology in the validation cohort. (A) MRI assessment versus postoperative invasion reference. (B) Endometrial biopsy versus final histologic reference. (C) Endometrial biopsy combined with MRI versus final risk stratification. (D) Nomogram versus final risk stratification.

Abbreviations: MI, myometrial invasion; CI, cervical invasion; EI, extrauterine invasion; G, grade; NEEC, non-endometrioid endometrial carcinoma.

Note: G1, G2, and G3 are histological grades of endometrioid endometrial carcinoma.
